# Salutatory

**Published:** 1869-05

**Authors:** 


### EDITORIAL DEPARTMENT.

*Salutatory.*—With the present number we commence Vol. III. of the Third Series of the *American Journal of Dental Science.* It is well known that this *Journal* is the oldest periodical in the world devoted to the propagation of the principles upon which Dental Surgery is founded; and it is not going beyond the truth to assert that this *Journal* was one of the most important agencies in the elevation of the dental profession to its present position of usefulness and influence. In proof of this we may refer to the account of the rise and progress of Dental Surgery in Harris's "Dictionary of Medical Terminology, Dental Surgery and the Collateral Sciences," the present edition of which was revised by the writer: "Until 1820 Dental Surgery had made but little progress in the United States; since that period its advance has been more rapid. In 1839 a periodical devoted to the interests of the profession, "The American Journal of Dental Science," was established. In February 1840 the Legislature of Maryland chartered the Baltimore College of Dental Surgery, and in July following, the American Society of Dental Surgeons was organized. The combined influence of the Journal, the College, and the American Society, gave an impetus to the Science which it had never before had, and contributed in an eminent degree, to the dignity and respectability of the profession."

The object of the editor in undertaking the duties and responsibilities devolving upon him, is to make the *Journal* an exponent of the status of dentistry, and a medium by which members of the profession can communicate their ideas to their fellows, and thus diffuse information and cultivate the habit of imparting it.

A generous co-operation from the members of the dental profession throughout the country is therefore respectfully solicited; and it will be the aim of the editor to make the *Journal* thoroughly practical, and to furnish its subscribers a faithful record of all improvements as fast as they are made known by their authors.

As the Journal is issued on the first of each month, its readers will be kept well informed as to the progress of the Science; the latest inventions will be duly noticed, and all suggestions impartially discussed, and every effort made to advance the best interests of the profession.

Communications are respectfully solicited on all subjects pertaining to the practice of dentistry, as well as reports of cases occurring in dental practice, proceedings of dental Societies, &c.

Want of cultivation in the habit imparting information, need be no excuse for neglect in making known important cases met with in practice, as due care will be taken that all communications appear in a proper form. All, therefore, may assist in advancing the interests of the profession, and at the same time add to their own reputation.

